# A comparative cross-sectional study of psychological distress, fatigue, and physical activity in patients with rheumatoid arthritis, systemic lupus erythematosus, and Sjögren's disease

**DOI:** 10.3389/fmed.2024.1507242

**Published:** 2025-01-07

**Authors:** Sonja Beider, Michael Stephan, Tabea Seeliger, Thomas Skripuletz, Torsten Witte, Diana Ernst

**Affiliations:** ^1^Department of Rheumatology and Immunology, Hannover Medical School, Hannover, Germany; ^2^Department of Psychosomatic Medicine, Hannover Medical School, Hannover, Germany; ^3^Department of Neurology, Hannover Medical School, Hannover, Germany

**Keywords:** depression, anxiety, rheumatoid arthritis, Sjögren's disease, systemic lupus erythematosus, physical activity, fatigue

## Abstract

**Introduction:**

Anxiety and depression are common in patients with rheumatic diseases, but their impact across conditions like rheumatoid arthritis (RA), systemic lupus erythematosus (SLE), and primary Sjögren's disease (SjD) is still not well understood. This study aims to compare depression, anxiety, and fatigue, and their effects on disease activity and physical activity in these conditions.

**Methods:**

From January 2019 to March 2021, patients with RA, primary SjD and SLE were assessed consecutively in a monocentric cross-sectional study at the rheumatology outpatient clinic of the Hannover Medical School. Standardized questionnaires were used to assess depression, anxiety, fatigue, disease activity, functional impairment, and physical activity in these patients.

**Results:**

Of 445 patients, 36.9% had RA, 32.8% SLE, and 30.3% SjD, with most being female (RA 76.2%, SLE 85.6%, SjD 87.4%). Depression (28.5%) and anxiety (31.2%) were common, particularly in SLE (28.8%) and SjD (36.3%) vs. RA (22%, *p* 0.002). Physical inactivity was higher in SLE (44.5%) and SjD (44.4%) than in RA (39.0%), especially in depressed patients (*p* 0.011). A significant proportion of patients retired early, especially in SLE (85%) and SjD (66%) vs. RA (49%, *p* 0.001). Disease activity correlated with psychological status (*p* < 0.05).

**Conclusions:**

Depression and anxiety are highly prevalent in RA, SLE, and SjD, particularly in SLE and SjD. The study highlights the need for early psychological evaluation and integrated care involving rheumatologists and mental health professionals to address these issues and improve physical and mental well-being.

## 1 Introduction

Depression has a major impact on health policy and health economics due to its complications and consequences (1). According to studies by the World Health Organization (WHO), the World Bank, and the European Brain Council (2), its social importance in Europe and Germany has been prioritized over other widespread diseases such as diabetes mellitus or coronary heart disease since the early 1990s. Therefore, depression is a major contributor to the global burden of disease in the general population (3). At the same time, depression is one of the most common comorbidities in patients with rheumatic diseases (4).

The prevalence of depression in chronic inflammatory diseases is estimated to be about two to four times higher than in the general population (5, 6). Numerous explanatory factors are cited for this, starting with the acceptance and personal confrontation with the diagnosis of a chronic, incurable disease, through the limitations on participation in social and life activities, to occupational or work disability as a result of the underlying disease and side effects of the medication. The loss of energy, pessimism and negative cognitive attitudes, and poor sleep quality, which are characteristic of depressive disorders, also impair the affected person's ability to cope with the illness (7, 8).

According to current research, inflammation takes a special place in the pathogenesis of depression. The hypothesis describes the influence of systemic inflammation in chronic autoimmune diseases on the brain and its function. Proinflammatory cytokines such as TNFα (tumor necrosis factor alpha), IL-6 (interleukin 6) and IL-1β (interleukin 1 beta), which circulate in the systemic blood flow in rheumatic diseases, influence the brain structures (9). Inflammation is thus able, through dysregulation and changes in the hypothalamus-pituitary-adrenal axis, to induce disease behavior corresponding to the neurovegetative characteristics of depression, e.g., lethargy, appetite, and sleep disorders (10). Current studies also assume that bidirectional associations between rheumatic diseases and depression exist through biological factors that involve common inflammatory pathways and that depression can serve as an independent risk factor for the development of rheumatoid arthritis (RA) (7).

Inflammation, pain and impaired functional capacity but also low mental status and depressive mood contribute to the fact that patients with rheumatic-inflammatory diseases tend to be less physically active than recommended. Over time, they run the risk of total physical inactivity (11, 12). In this context, fatigue, which is recognized as a relevant clinical problem, is often discussed (13, 14). Physical activity can have a positive benefit on fatigue (15). It also contributes to the prevention of many relevant diseases such as stroke, diabetes, obesity, and oncological and cardiovascular diseases (16–18). For rheumatoid arthritis, there are already existing recommendations for physical activity written by EULAR (European League Against Rheumatism) suggesting a daily moderate physical activity of at least 30 min (19).

The aim of the study was to understand how psychological factors such as depression, anxiety, and fatigue affect patients with RA, systemic lupus erythematosus (SLE), and primary Sjögren's disease (SjD). While these mental health issues are known to be prevalent in rheumatic diseases, there was limited research comparing their impact across these three specific conditions. Furthermore, the relationships between psychological factors, disease activity, and physical activity levels had not been comprehensively explored. By addressing these gaps, the study aimed to provide insights that could inform tailored interventions to improve the mental and physical well-being of patients with these chronic diseases.

## 2 Methods

### 2.1 Data collection

Patients with RA, SLE, and SjD, who visited the rheumatological outpatient clinic of the Hannover Medical School between January 2019 and March 2021 were asked to take part in this study. The inclusion criteria included the diagnosis of RA, SLE or SjD as the primary diagnosis, the ability to provide informed consent, and being of legal age. Patients with written consent were consecutively enrolled, and the study was approved by the local Ethics Committee (8179_BO_S_2018). In the data collection process, anthropometric parameters including age, gender, weight, height, and body mass index (BMI) were measured. Additionally, data on dietary habits, alcohol consumption, and smoking behavior were gathered. Participants were excluded if they had more than one underlying rheumatic diagnosis.

### 2.2 Indices

The following standardized questionnaires were used in the data collection: Beck Depression Inventory Second Edition (BDI II) and the Hospital Anxiety and Depression Scale (HADS) for the assessment of anxiety and depressive symptoms, the Multidimensional Assessment of Fatigue (MAF) for the identification of fatigue, the European League Against Rheumatism (EULAR) Sjögren's syndrome disease activity index (ESSDAI), and the EULAR Sjögren's Syndrome Patient Reported Index (ESSPRI), the Hannover Functional Questionnaire (FFbH) for the assessment of functional capacity and the International Physical Activity Questionnaire short form (IPAQ-SF) to determine the level of physical activity.

### 2.3 Statistical analysis

Statistical analyses were performed using SPSS software (version 27, IBM Corporation, 1989–2020). Quantitative variables were presented as mean and standard deviation (SD), while categorical variables were expressed as frequency (n) and percentage (%). Pearson's correlation was used to analyze relationships between variables, followed by partial correlation to control for age as a confounding factor. The normality of data distribution was assessed using the Kolmogorov-Smirnov test. Both parametric and non-parametric tests were applied to evaluate the distribution of risk factors across the study groups.

Significant differences were analyzed using the Chi-square test, Mann-Whitney U test, and Kruskal-Wallis test. Median values were compared using the Hodges–Lehmann test, providing two-sided 95% confidence intervals. Missing values for categorical variables were excluded from the analysis. To address potential Type I errors due to multiple comparisons, the Bonferroni correction was applied. A two-sided significance level of 0.05 was applied for all tests.

## 3 Results

### 3.1 Demographics

A total of 445 patients were included in the study. 164 (36.9 %) patients had RA, 146 (32.8 %) had SLE, and 135 (30.3 %) had SjD. The mean age of the patients was 53.6 ± 14.0 years (ranging from 19 to 85 years) with the SLE patients being slightly younger on average at 48.6 years. The proportion of affected females was 82.7% (368) in total, RA 76.2 %, SLE 85.6 %, SjD 87.4 %. The median disease duration of the patients was 110 months (ranging from 1 to 641 months). Patient characteristics according to underlying disease are detailed in Table 1.

**Table 1:** Patient characteristics by diagnosis.

**Parameter**		**In total**	**SjD**	**SLE**	**RA**	***p***
***n***		**445**	**135**	**146**	**164**	
Age, y, mean ± SD (min-max)		53.6 ± 14.0 (19–85)	54.9 ± 15.0 (21–85)	48.6 ± 12.2 (19–73)	56.9 ± 13.4 (21–84)	< 0.001
Male, *n* (%)		77 (17.3)	17 (12.6)	21 (14.4)	39 (23.8)	0.021
Female, *n* (%)		368 (82.7)	118 (87.4)	125 (85.6)	125 (76.2)	
Disease duration, mo, mean ± SD		129.2 ± 105.3	72.5 ± 74.2	153.6 ± 101.7	154.3 ± 112.4	< 0.001
BMI, kg/m^2^, mean ± SD (min-max)		26.2 ± 5.6 (14.5–50.0)	26.3 ± 5.7 (15.5-44.1)	25.7 ± 6.1 (14.5-50)	26.5 ± 4.9 (18–42.5)	0.110
Nutritional status according to BMI, *n* (%)	BMI < 18.5 kg/m^2^	15 (3.4)	7 (5.2)	7 (4.8)	1 (0.6)	0.025
	BMI 18.5–24.9 kg/m^2^	203 (45.6)	57 (42.2)	77 (52.7)	69 (42.1)	
	BMI 25–29.9 kg/m^2^	127 (28.5)	37 (27.4)	36 (24.7)	54 (32.9)	
	BMI > 30 kg/m^2^	100 (22.5)	34 (25.4)	26 (17.8)	40 (24.4)	
Eating habits, *n* (%)	No restrictions	412 (92.6)	126 (93.3)	136 (93.2)	150 (91.5)	0.793
	Vegetarian	32 (7.2)	9 (6.7)	9 (6.2)	14 (8.5)	
	Vegan	1 (0.2)	0	1 (0.7)	0	
Smoking, *n* (%)	Never smoked	260 (58.4)	85 (63.0)	78 (53.4)	97 (59.1)	0.126
	Ex-smoker	111 (24.9)	38 (28.1)	40 (27.4)	33 (20.1)	
	Smoker	74 (16.6)	12 (8.9)	28 (19.2)	34 (20.7)	
Alcohol, g per Week, *n* (%), mean ± SD		170 (38.2), 30.7 ± 34.5	63 (46.7), 24.4 ± 30.1	49 (33.6), 35.1 ± 43.0	58 (35.4), 33.9 ± 30.7	0.137
No alcohol, *n* (%)		275 (61.8)	72 (53.3)	97 (66.4)	106 (64.6)	0.050
Retirees, *n* (%)	In total	185 (41.6)	61 (45.2)	48 (32.8)	76 (46.3)	< 0.001
	Regular retirement	67 (15.1)	21 (15.6)	7 (4.8)	39 (23.8)	
	Early retirement	118 (26.5)	40 (29.6)	41 (28.1)	37 (22.6)	
Disability, *n* (%)	> 50 %	148 (33.3)	43 (31.9)	51 (34.9)	54 (32.9)	0.127
	≤ 50 %	102 (22.9)	41 (30.4)	39 (26.7)	22 (13.4)	
	No disability	195 (43.8)	51 (37.8)	56 (38.4)	88 (53.7)	
Depression (BDI II ≥ 14), *n* (%)		127 (28.5)	49 (36.3)	42 (28.8)	36 (22)	0.002
BDI II, mean ± SD		10.8 ± 9.6	11.8 ± 9.3	12.1 ± 10.7	8.9 ± 8.5	
Anxiety disorders (HADS A ≥ 8), *n* (%)		139 (31.2)	49 (36.3)	52 (35.6)	38 (23.2)	0.020
HADS A, mean ± SD		5.9 ± 4.3	6.2 ± 4.2	6.4 ± 4.6	5.2 ± 4.1	0.020
Depression (HADS D ≥ 8), *n* (%)		110 (24.7)	35 (25.9)	40 (27.4)	35 (21.3)	0.434
HADS D, mean ± SD		5.0 ± 4.2	5.1 ± 4.2	5.4± 4.3	4.6 ± 4.2	0.224
Fatigue (GFI ≥ 20), *n* (%)		285 (64)	87 (64.4)	107 (73.3)	91 (55.5)	
Fatigue (GFI), mean ± SD		24.2 ± 11.7	24 ± 11.9	26.9 ± 11.4	21.9 ± 11.5	
Physical activity, IPAQ, *n* (%)	Low	189 (42.5)	60 (44.4)	65 (44.5)	64 (39)	0.724
	Moderate	147 (33.0)	44 (32.6)	43 (29.5)	60 (36.6)	
	High	109 (24.5)	31 (23)	38 (26)	40 (24.4)	
Physical activity	MET, mean ± SD	2,126 ± 5,082	2,492 ± 3,610	3,028 ± 7,399	2,353 ± 3,273	0.718
Functional capacity	FFbH < 60 %, *n* (%)	88 (19.8)	25 (18.5)	22 (15.1)	41 (25.0)	
Functional capacity	FFbH, mean ± SD	77.5 ± 21.7	78.9 ± 19.8	80.7 ± 20,6	73,6 ± 23,5	0.013

Mean average, SD standard deviation, n number of participants, min-max minimum**–**maximum, y years, mo month, g grams, BDI II Beck Depression Inventory II, HADS Hospital Anxiety and Depression Scale, GFI Global Fatigue index, IPAQ International Physical Activity Questionnaire, MET Metabolic Equivalent Task, BMI Body Mass Index, FFbH Hannover Functional Questionnaire.

### 3.2 Depression and anxiety

In the overall cohort, 127 patients (28.5 %) were found to have depression based on a BDI-II score of ≥14. Among these patients, 13.9 % (62) showed mild, 7.4 % (33) exhibited moderate, and 7.2 % (32) suffered from severe depression. Patients classified with mild depression exhibited an average BDI-II score of 16.2, whereas those diagnosed with severe depression demonstrated a notably higher mean score of 35.4. Out of all the depressive symptoms that were queried as part of the BDI II survey, lack of energy and tiredness (fatigue) were particularly important; around $\frac{3}{4}$ of all patients reported these symptoms. More than 50 % of all patients reported other somatic symptoms such as concentration and sleep disorders, loss of pleasure and loss of libido.

Patients with SLE and SjD were more likely to be affected by depression than those with RA. About one-fifth of all RA patients showed symptoms of depression (36/164, 21.9 %). This proportion was higher in SLE patients (42/146, 28.8 %), and even higher in SjD patients (49/135, 36.3 %), p 0.002. The HADS-D questionnaire revealed that 110 patients (24.7%) in the overall cohort exhibited depression, based on a cut-off score of HADS ≥ 8. The average HADS-D score for patients with borderline depression was 8.8, while the average score for patients with pathological depression was 13.5. As the BDI II and HADS D depression scores exhibited a strong correlation in the study cohort (*p* < 0.001), redundancy in the description was avoided by solely referring to the BDI II score in the analysis presented in the following manuscript.

In the entire cohort, a significant number of 139 (31.2 %) patients indicated the presence of anxiety symptoms based on the criteria of HADS-A ≥ 8. For patients with pathological anxiety, the average HADS-A score was 13.2. Among RA patients, 23.1 % (38/164) reported anxiety issues, while SLE and SjD patients experienced a notably higher prevalence of anxiety symptoms: 35.6 % (52/146) for SLE and 36.3 % (49/135) for SjD, respectively (*p* 0.020). Differences in the parameters assessed were observed between patients with and without depression (Table 2). Although comparable in age and gender distribution, the two patient groups demonstrated noticeable differences in several other variables. Specifically, patients with depression exhibited a comparatively shorter mean duration of illness (103 vs. 140 months, *p* < 0.001), higher levels of fatigue (GFI of 32.8 vs. 20.8, *p* < 0.001), greater functional impairment (FFbH score of 68.4 % vs. 81.2 %, *p* < 0.001), and lower levels of physical activity (1,975 weekly MET-min vs. 2,873 MET-min, *p* 0.016).

**Table 2:** Parameters related to depression.

**Parameter**	**BDI II (**≥**14)**		***p***	**HADS D (**≥**8)**		***p***
	**Depression**	**No depression**		**Depression**	**No depression**	
Patients, *n*	127	318		110	335	
Age, y, mean ± SD	52.5 ± 13.2	54 ± 14.2	0.142	52.0 ± 13.4	54.1 ± 14.1	0.075
Female, *n* (%)	112 (88.2)	256 (80.5)	0.056	92 (83.6)	276 (82.4)	0.765
Disease duration, mo, mean ± SD	103.2 ± 95.3	139.6 ± 107.5	< 0.001	116.4 ± 96.8	133.4 ± 107.8	0.159
BDI II, mean ± SD	23.0 ± 8.8	6.0 ± 4.0	< 0.001	22.0 ± 10.9	7.2 ± 5.5	< 0.001
HADS D, mean ± SD	9.5 ± 3.9	3.2 ± 2.7	< 0.001	11.0 ± 3.0	3.0 ± 2.3	< 0.001
HADS A, mean ± SD	10.4 ± 3.8	4.1 ± 3.0	< 0.001	10.3 ± 4.0	4.4 ± 3.3	< 0.001
Fatigue, GFI, mean ± SD	32.8 ± 8.7	20.8 ± 11	< 0.001	32.0 ± 9.3	21.6 ± 11.3	< 0.001
Functional capacity, FFbH, mean ± SD	68.4 ± 22.9	81.2 ± 20.1	< 0.001	66.9 ± 23.2	81.1 ± 20.0	< 0.001
Physical activity, MET, mean ± SD	1,975.4 ± 3,681.9	2,872.7 ± 5,528.1	0.016	1,824.7 ± 2,899.9	2,876.6 ± 5,596.3	0.005

Mean, average; SD, standard deviation; n, number; y, years; mo, month; BDI II, Beck Depression Inventory II; HADS, Hospital Anxiety and Depression Scale; GFI, Global Fatigue Index; MET, Metabolic Equivalent Task; FFbH, Hannover Functional Questionnaire.

The findings of the study revealed a significant co-occurrence of anxiety symptoms among patients with depression. Specifically, a vast majority of patients with depression, comprising 78 % of the cohort (99), also displayed symptoms of anxiety (*p* < 0.001). In contrast, a relatively small subset of the patient cohort, consisting of 22 % (28), demonstrated symptoms of depression in isolation without any evidence of comorbid anxiety disorder.

### 3.3 Physical activity, functional capacity, and fatigue

Regarding physical activity, the patient cohort was stratified into three groups: 42.5 % (189) displayed low levels of physical activity, 33.0 % (147) showed moderate levels, and 24.5 % (109) exhibited high levels. Patients with high physical activity levels demonstrated an average weekly MET-min value of almost 7.400 higher than those with low levels of physical activity. The distribution varied among the patient groups: While 39 % (64/164) of RA patients had low physical activity, this proportion was higher among SjD patients with 44.4 % (60/135) and among SLE patients with 44.5 % (65/146). Furthermore, nearly half of the 319 patients who responded to the daily sitting duration query reported sitting for over 6 h each day.

The level of physical activity showed a notable difference in distribution between patients with and without psychiatric disorders. The proportion of individuals with low levels of physical activity was higher in the group of patients with depression, 63/127, 49.6 % vs. 126/318, 39.6 % (*p* 0.011). While 38.9 % (14/36) of RA patients with depression exhibited low physical activity, this proportion was higher among patients with SLE or SjD. Among SjD patients with depression, 51 % (25/49) showed a low physical activity level, and among SLE patients with depression, the proportion was 57.1 % (24/42), indicating that more than half of these patients were inactive. The mean weekly number of MET-min showed a significant difference between patients with and without depression, particularly among SLE patients, 2,138 vs. ,3388 (*p* 0.008).

A relevant functional impairment (FFbH < 60 %) was found in 19.6 % of all patients (87) with a mean FFbH value of 41 %. Patients with an unrestricted functional capacity had a mean FFbH value of around 93 %. RA patients had a higher proportion of individuals with functional impairment (41/164, 25 %) than SjD or SLE patients (*p* 0,013).

Fatigue was observed in 64.0 % of patients (285/445) who exhibited a GFI score >20. Patients with fatigue had a significantly higher average GFI score of 30.7 compared to those without fatigue, who had an average GFI score of 12.5. The GFI score was found to be negatively associated with physical activity levels. Notably, the average GFI score was significantly higher in patients with lower physical activity levels (GFI 26.2) compared to those with moderate to high physical activity levels (GFI 22.7, p < 0.001). SLE and SjD patients experienced more fatigue (mean GFI SjD 24, SLE 27) than RA patients (mean GFI 22). Among SLE patients, fatigue was most prevalent at 73.3 % (107/146), *p* 0.001.

### 3.4 Premature retirement

The study cohort comprised 185 retired patients (41.6 %), including 67 on old-age pension (36.2 %) and 118 patients who retired prematurely (prior to the age of 65) due to disease-related disability (63.8 %). Especially in the case of SLE and SjD, the proportion of patients that retired prematurely was high: 41/48, 85 % with SLE and 40/61, 66 % with SjD. In contrast, among pensioners with RA, the proportion of early retirees was 49 %, 37/76 (*p* 0.001). The patients who retired prematurely due to illness were significantly more likely to suffer from depression (mean BDI II score 12.6 vs. 7.8, *p* 0.003). This association was particularly prominent in SLE patients (14.4 vs. 7.4). Moreover, early retirement status was found to be associated with the severity of depression (*p* = 0.008).

The study also investigated the reasons for premature retirement among the patients. All SjD patients (40/40) indicated their underlying disease as the cause of their early retirement, while 10.8 % (4/37) of RA patients and 31.7% (13/41) of SLE patients were retired prematurely for other reasons.

### 3.5 Disease activity

The disease activity was determined for all diagnoses using the indices defined according to EULAR criteria. For RA patients, the DAS28 value was used to determine disease activity. More than half of all RA patients (61 %, 100/164) had low, one third of the patients (32.9 %, 54/164) had a medium and 6.1 % (10/164) high disease activity level. This distribution is reflected in the mean DAS28 score of 2.9. The psychological status of RA patients was found to be dependent on disease activity. The average DAS28 score was distributed differently among patients with and without depression (p 0.002). Differences were observed between individuals with and without depression in the calculation of DAS28 scores based on parameters such as painful joints (3.5 vs. 1.3) and the subjectively determined patient's state of disease (visual analog scale, VAS), 57.1 vs. 42.9. In patients with SjD, disease activity was assessed using ESSDAI and ESSPRI. ESSDAI data was available for 120 out of 135 SjD patients, with one-fifth of these patients showing high disease activity and one-third showing moderate disease activity. The psychological status of SjD patients showed no association with disease activity as measured by ESSDAI (p 0.869). However, there was a significant difference in average ESSPRI scores between patients with and without anxiety (6.1 vs. 4.5, *p* < 0.001) or depression symptoms (6.2 vs. 4.4, *p* < 0.001). In SLE patients, disease activity as measured by SLEDAI showed no association with depression but was associated with anxiety disorders (3.1 vs. 1.6, *p* 0.032).

## 4 Discussion

### 4.1 Differences regarding mental health

We found a high prevalence of depression and anxiety disorders among patients with RA, SjD, and SLE. These rates are higher than the corresponding numbers in the general population. While the prevalence of depression in Germany is 9.2% and 6.6% on average across Europe (20), 28.5% of our cohort suffered from depressive symptoms. The fact that patients with rheumatic diseases are more frequently affected by depression and anxiety disorders is well known (21, 22). The prevalence of depression in chronic inflammatory diseases is estimated to be approximately two to four times higher than in the general population (5, 23). Anyfanti et al. report a prevalence of depression of 21.8% and anxiety disorders of 30.8% in these patients (24).

Our study shows that depression and anxiety disorders were more frequently observed in patients with collagenoses compared to patients with RA. SLE patients were more often affected by depression than RA patients, and the proportion of patients with depression was even higher among those with SjD. The literature confirms the high prevalence of depression in patients with SjD; Cui et al. report numbers of up to 36.9% in their SjD cohort (25). Furthermore, the authors demonstrate a prevalence of anxiety disorders of 33.8%. In our cohort, SjD patients exhibited anxiety disorders even more often (36.3%). Similarly high rates were observed in our SLE patients. In contrast, the proportion of RA patients suffering from anxiety disorders was 21.9%, which is comparable to numbers in the general population. In the German general population, the annual prevalence of anxiety disorders is 21.4% among women and 9.3% among men (26, 27).

In our cohort, patients with depression had a shorter disease duration concerning their underlying condition compared to individuals without depression. Callhoff et al. report that the risk of developing depression is highest in RA patients within the first 5 years of the disease (28).

### 4.2 Differences regarding disease activity

Disease activity in RA patients differed significantly between patients with and without depression. More precisely, the average DAS28 score in patients with depression was approximately 25% higher than for those without depression. This correlation has been noted in previous studies (29–31). In our cohort, two domains of the DAS28 played a major role: the number of tender joints and the patient's self-assessment of their disease status (VAS). Interestingly the objective measurement of CRP did not play a significant role, which suggests pain as one of the leading factors for depression in RA patients. This finding also aligns with a study by Matcham et al. who report in a cohort of 56 RA patients, that symptoms of depression and anxiety have implications for disease activity, as measured via the DAS28, primarily due to subjective features such as tender joints and patient global assessment (32).

Regarding the disease activity of our SjD patients, we found that ESSPRI parameters highly significantly correlated with both anxiety disorders and depression. This result was primarily driven by the domains of pain and fatigue, which significantly differed between SjD patients with and without depression. The association of pain symptoms with anxiety, fatigue, and depression is well-documented across various disease entities, including oncology (33). Our findings align with recent data on SjD patients. For example, Sandoval-Flores et al. reported a correlation between unsatisfactory symptom state (defined as ESSPRI ≥ 5) and depression (34). This underscores the importance of subjective parameters in assessing disease activity in SjD. Furthermore, previous data from our own research group revealed that a representative subgroup of SjD patients reported the highest pain and fatigue scores as well as the most subjective dryness, while not showing any severe objective abnormalities (35).

Since pain is a significant factor for depression in both diseases, it should be thoroughly addressed during patient history-taking. Analgesic treatment should be prioritized, particularly when no objective signs of disease activity are evident.

### 4.3 Differences regarding physical activity, fatigue, and functional capacity

Almost half of the entire cohort reported low physical activity. These patients, regardless of their diagnosis, were significantly more likely to suffer from anxiety disorders, depression, and fatigue. Nearly one-fifth showed a significant functional impairment (FFbH < 60%). RA patients were more frequently affected by functional impairment than SjD and SLE patients. However, the proportion of individuals with low physical activity was lower among RA patients compared to SjD and SLE patients, particularly among those with depression. Numerous studies have demonstrated that rheumatic diseases are associated with reduced functional capacity and physical activity (36–39). In our cohort, some patients exhibited both characteristics, with the proportion being higher among RA patients compared to SjD and SLE patients.

Among patients with high disease activity, the proportion of individuals with low physical activity varied depending on the disease activity index. Approximately one-sixth of RA patients with moderate to high disease activity (DAS28 ≥ 3.6), one-seventh of SLE patients with high disease activity (SLEDAI ≥ 5), and one-fourth of SjD patients (ESSPRI ≥ 5) exhibited low physical activity.

Fatigue is frequently discussed as a significant clinical issue contributing to reduced physical activity (13, 14). Fatigue is a common symptom in inflammatory rheumatic diseases; ~40–70% of RA patients (40, 41) and up to 90% of SLE patients report severe fatigue (42, 43). In SjD, fatigue is one of the most frequently reported complaints after sicca symptoms, affecting up to 85% of patients (44). Fatigue is a multidimensional and burdensome symptom for RA patients, hindering their improvement, it seems to be far more important than other factors for difficult-to-treat RA development (45). In our cohort, fatigue was also commonly reported, with 64% of all patients having a GFI ≥ 20. The incidence was highest among SLE patients, consistent with the literature, at 73.3%. Fatigue correlated with both anxiety and depressive symptoms, as well as with the level of physical activity. Fatigue and mental health disorders may be directly associated with the disease itself, particularly in SLE (46), or may reflect insufficient coping mechanisms. Psychological support can improve disease coping and acceptance (47).

Social tools and emotional support are independent factors influencing the severity of depression in rheumatic diseases (48). Emotional support, for instance, has been shown to positively impact the severity of depressive symptoms in RA, independent of disease activity (49). Various forms of such support are already offered by statutory health insurers, such as psychosomatic group therapies, although their full potential remains untapped. According to the BARGRU study, the number of offered group therapies has increased annually (49,246 patients in 2018 compared to 37,001 in 2016). However, there is still a nationwide shortfall in availability (50).

Almost half of the respondents reported spending more than 6 h a day sitting. However, daily sitting time does not necessarily correlate with overall physical activity; more than half of the patients who spent over 6 h sitting daily reported moderate to high physical activity. The dependence of sitting behavior on physical activity levels in rheumatologic patients is generally questioned in the literature (51). Our own data also supported this, showing no correlation between daily sitting time and physical activity in a study of 114 RA patients (52).

Physical activity has been shown to have a positive impact on health, contributing to the prevention of various conditions such as cancer, stroke, diabetes, cardiovascular diseases, and obesity (16–18). Furthermore, physical exercise not only helps reduce disease activity and fatigue but also alleviates pain, enhances functional capacity, and positively influences the mental health of rheumatologic patients (13, 36, 53, 54).

EULAR recommends leveraging the positive effects of physical activity on the progression of rheumatic diseases and incorporating it as part of standard therapy. The minimum recommended level of physical activity is at least 30 minutes of moderate activity daily or a weekly total of 500 MET-minutes (19). However, caution should be exercised with physical activity, especially for patients with chronic fatigue syndrome or myalgic encephalomyelitis (CFS/ME), to avoid potential adverse effects (55).

Non-pharmacological treatments can include physiotherapy or functional training, both of which have the potential to improve mental well-being (56). In our patient cohort, less than half of RA patients received non-pharmacological therapy, and this proportion was even lower among patients with collagen diseases.

### 4.4 Impact on employment

Rheumatic diseases often lead to significant limitations in professional life, eventually resulting in complete work incapacity (57). Long-term studies show a cumulative prevalence of work disability in RA patients of 10% after 1 year, rising to 90% after 30 years of disease progression (58). Patients with collagen diseases are also at higher risk of work disability compared to the general population. For SLE a cumulative prevalence of work disability of 23% has been reported (59). Mandl et al. found that 26% of SjD patients are disabled at diagnosis, with this proportion increasing to 37% after 12 months and 41% after 24 months (60).

In our cohort, work disability was assessed through early retirement data. We observed the same trend as reported in the literature: collagenoses contribute more significantly to work disability than RA. One in five RA patients reported early retirement, while one in three patients with collagen diseases did. When asked about reasons for early retirement, all SjD patients cited their underlying disease, whereas some RA and one-third of SLE patients attributed their early retirement to other causes. It is well-established that depression is a predictor of work disability in RA patients (28). However, it is likely that psychological factors, such as depression, also play an increasing role in work disability among SLE patients.

### 4.5 Limitations

The monocentric nature of this study limits the robustness of the data. Our cohort consisted of patients from a university clinic, leading to an overrepresentation of individuals with severe disease courses. Additionally, participation was voluntary, potentially introducing selection bias, as patients more open to these topics may have been more likely to participate. Moreover, the individual groups were not matched for age and gender, which affects the comparability of certain findings. The results were derived solely from questionnaire analyses, making them susceptible to individual sensitivities. To enhance comparability in future research, incorporating cognitive function tests would be beneficial.

### 4.6 Conclusions and perspective

Mental disorders such as depression and anxiety were highly prevalent in our cohort of RA, SjD, and SLE patients. Depression and anxiety played a more significant role in patients with collagenoses and may contribute to the development of disease-related work disability. These findings emphasize the importance of early evaluation of the psychological status in patients with rheumatic diseases. Questions regarding mental health should be routinely included in the initial medical history interview. To improve disease coping and acceptance, psychological support, such as psychosomatic group therapies, should be more frequently utilized and recommended by attending physicians. Given the relatively high proportion of patients with low physical activity in our cohort, there is potential for improvement in the current care model. Increased use of physiotherapy and functional training would be advisable to promote better physical activity levels. Ongoing monitoring, not only of disease-specific activity but also of mental health, is essential for ensuring optimal and holistic care of chronically ill patients. At the same time, a multidisciplinary approach involving rheumatologists, psychiatrists, neurologists, psychologists, as well as physiotherapists and pain therapists, would be beneficial for improving therapeutic outcomes in patients with rheumatic diseases who present with mental health issues.

## Data Availability

The raw data supporting the conclusions of this article will be made available by the authors, without undue reservation.
